# The "rapid atrial swirl sign" for assessing central venous catheters: Performance by medical residents after limited training

**DOI:** 10.1371/journal.pone.0199345

**Published:** 2018-07-16

**Authors:** Peter Korsten, Eirini Mavropoulou, Susanne Wienbeck, David Ellenberger, Daniel Patschan, Michael Zeisberg, Radovan Vasko, Björn Tampe, Gerhard A. Müller

**Affiliations:** 1 Department of Nephrology and Rheumatology, University Medical Center Goettingen, Goettingen, Germany; 2 Department of Gastroenterology and Gastrointestinal Oncology, University Medical Center Goettingen, Goettingen, Germany; 3 Institute for Diagnostic and Interventional Radiology, University Medical Center Goettingen, Goettingen, Germany; 4 Department of Medical Statistics, University Medical Center Goettingen, Goettingen, Germany; University of Bern, SWITZERLAND

## Abstract

**Rationale:**

Central venous catheter (CVC) placement is a standard procedure in critical care. Ultrasound guidance during placement is recommended by current guidelines, but there is no consensus on the best method for evaluating the correct CVC tip position. Recently, the “rapid atrial swirl sign” (RASS) has been investigated in a limited number of studies.

**Objectives:**

We performed a prospective diagnostic accuracy study of focused echocardiography for the evaluation of CVC tip position in our medical ICU and IMC units.

**Methods:**

We performed a prospective diagnostic accuracy study in 100 patients admitted to the Intensive Care Unit and Intermediate Care Unit at our center. The first 10 subjects were assessed by one staff physician investigator (reference cohort), the remaining 90 patients by different residents (test cohort). All patients received a post-procedural chest radiograph (CXR) as gold standard. CVC placement was assessed with focused echocardiography performed by residents after a short training session. A rapid opacification of the right atrium (RASS) after injection of 10 mL of normal saline was regarded as “positive”, flush after more than two seconds was defined as “delayed”, no flush was a “negative” test result.

**Measurements and main results:**

Overall sensitivity of the RASS was 100% (95% CI 73.54–100%), specificity was 94.32% (CI 87.24–98.13%). Positive and negative predictive values were 70.59% (CI 44.04–89.09%) and 100% (CI 95.65–100%), respectively. Median time for echocardiographic testing was 5 minutes (1–28) in the whole cohort, CXRs were available after 49.5 minutes (13–254). Interrater agreement of the RASS was 0.77 (Cohen’s kappa), Measurement of CVC tip position was not different between two observers. Test characteristics were similar among differently experienced residents.

**Conclusions:**

Presence of the RASS by focused echocardiography showed excellent sensitivity and specificity and was equally performed by residents after minimal training. In patients with a positive RASS, routine CXR can be safely omitted, reducing time, costs and radiation exposure. A negative RASS should lead to a search for misplaced catheters.

**Clinical trial registration:**

The study was registered with www.clinicaltrials.gov (NCT02661607).

## Introduction

Central venous catheter (CVC) placement is a standard procedure in emergency and critical care environments. The recently published guidelines of the European Federation of Societies for the use of Ultrasound in Medicine and Biology (EFSUMB) recommend the use of ultrasound (US) for placement of CVCs, which has been shown to significantly reduce the risk of complications, such as inadvertent arterial puncture, pneumothorax or bleeding [1]. The use of US has also been found to facilitate subclavian vein (SV) or axillary vein (AV) cannulation, which is often not performed because of the more technically more demanding approach [2,3].

While the use of US is considered standard of care for placement of CVCs, there is no consensus on the best method for the evaluation of proper placement and tip localization. Therefore, in most intensive care units (ICU) or intermediate care units (IMC), post-procedural chest radiography (CXR) is still considered the gold standard for the assessment of correct CVC tip position. Other techniques include US guidewire visualization in the inferior vena cava or within the right atrium [4] and electrocardiographic recognition of altered P-waves [5,6]. In the developing world, techniques based on clinical bedside tests without specialized equipment have been investigated [7]. However, these techniques are technically more cumbersome, require special equipment or are potentially hazardous placing patients at risk for arrhythmias, injury to vessels or cardiac structures.

Various less invasive techniques, including contrast-enhanced US (CEUS), have been described [8]. In 2010, Prekker *et al*. reported the use of the “saline flush” method with the “rapid atrial swirl sign” (RASS) in order to assess proper CVC tip placement [9]. Subsequent studies have demonstrated its usefulness in adult [10] and pediatric [11] patients. However, the number of prospective trials on RASS for the assessment of CVC tip placement is limited.

We performed a prospective diagnostic accuracy study of focused echocardiography for the evaluation of CVC tip position in our medical ICU and IMC units. The primary endpoint was to evaluate the performance of focused echocardiography compared with conventional CXR in two independent cohorts. Ideally, such a procedure should be easy to learn and perform, therefore, we also evaluated the performance of all residents assigned to the ICU and IMC during the study period as a secondary endpoint.

## Methods

### Setting, study population and study criteria

**Fig 1:**
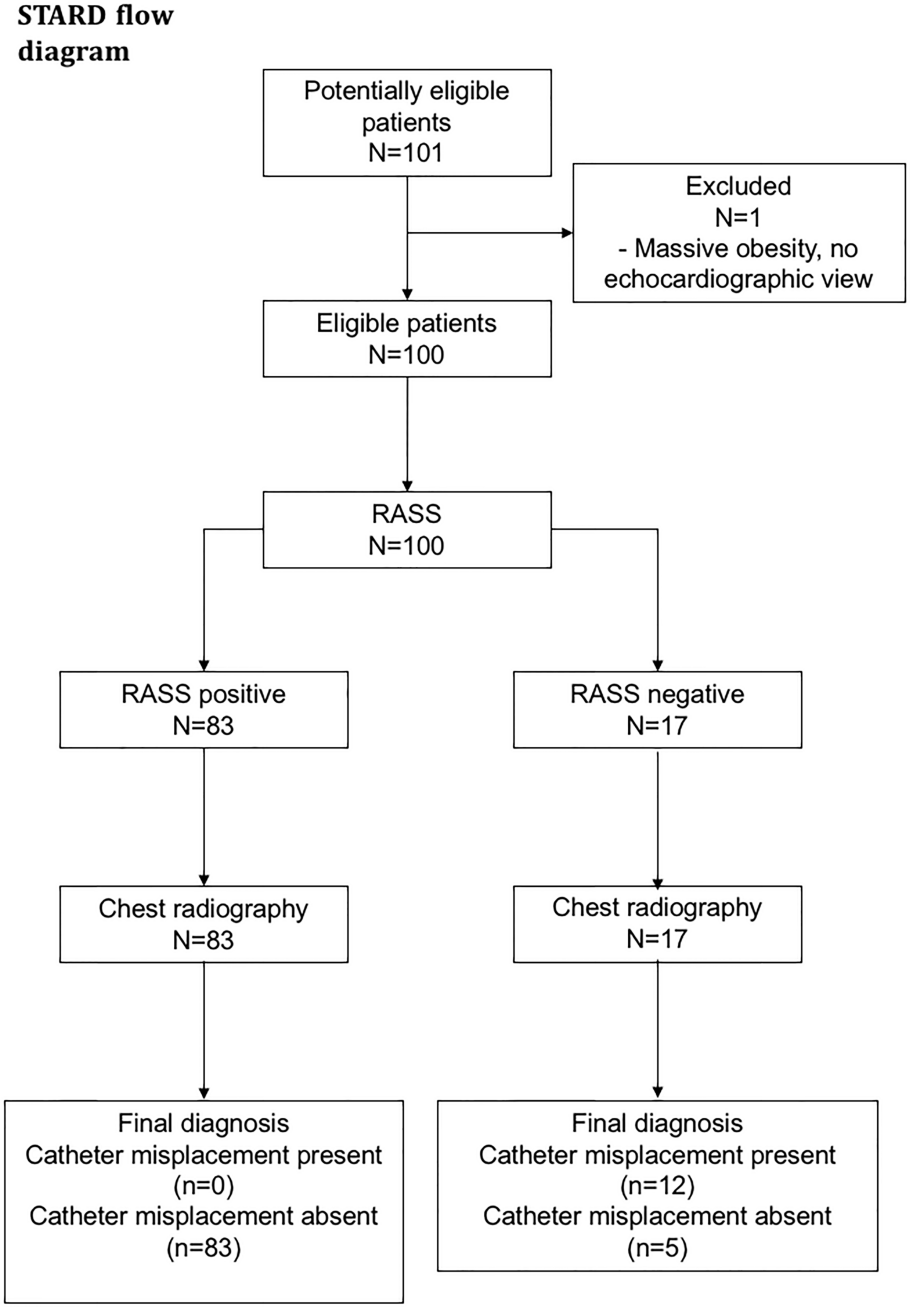
Patient disposition according to the STARD (Standard reporting in Diagnostic accuracy studies) flowchart. **Abbreviations used**: RASS: rapid atrial swirl sign.

The trial was conducted in a convenience sample of patients admitted to the medical ICU and IMC of the Department of Nephrology and Rheumatology at University Medical Center Goettingen, Germany from October 2014 to April 2016. The disposition of included patients is shown in Fig 1.

Inclusion criteria consisted of: ventilated or non-ventilated adult patients admitted to either the ICU or IMC requiring a central venous catheter for medical reasons and written informed consent signed by the patient or the patient's legally authorized representative (for unconscious patients). Only patients with CVCs placed in the subclavian or internal jugular veins were included in the study.

Exclusion criteria were: age under 18 years, inability/unwillingness to provide written informed consent, inability to perform the index test due to anatomical limitations and placement of femoral central venous catheters.

We decided firstly to test the study protocol/procedure in 10 patients (referred to as the "reference cohort") with echocardiography performed by only one trained investigator (EM). To further test performance by medical residents, focused echocardiography was performed by a total of 20 different residents of post-graduate years (PGY) one to six in 90 additional patients ("test cohort"). Sample size estimation is given in the supporting information file (data in **S1 Text**).

### CVC placement and assessment on chest radiographs

Residents assigned to the ICU or IMC during their rotation inserted central venous catheters according to standard local practice (US-guided for internal jugular vein placement, US-guided or according to the landmark method for subclavian vein placement as per individual preference, using aseptic Seldinger technique). Residents were required to have placed at least 20 CVCs or were supervised by a senior resident or attending physician with experience of more than 100 placed CVCs. These numbers were chosen arbitrarily and represent our experience with residents or senior faculty, which we consider appropriately capable of placing/supervising CVCs.

We used three-, four- or five-lumen catheters (ARROWg+ard Blue with Blue FlexTip®, Four- and Five-Lumen LOGICATH™ Kit, Teleflex Inc., Morrisville, North Carolina, USA) at the discretion of the resident performing catheterization. After CVC placement, radiology service was called to perform conventional anterior-posterior chest radiography (the reference standard) in all patients as routinely performed at our institution. The time required until CXRs were available for evaluation was recorded.

### CVC tip position

All routinely performed post-procedural chest radiographs were read by the person responsible for placing the catheter (the interventionist) and by a radiologist as it is usual practice at our institution. Misplaced catheters were corrected when deemed necessary by the interventionist. Additionally, all CXRs were read by one investigator (EM) and one radiologist (SW) and catheter tip position measured in relation to the carina. CVC tip position in relation to the carina on CXRs were evaluated independently by one investigator (EM) and by a board-certified radiologist (SW). The radiologist was blinded to all other study procedures except for the evaluation of the CVC tip position on radiographs. CVC tip positions were classified as projecting into zone A (vena cava superiorly to the carina), B (vena cava inferiorly to the carina), C (innominate vein) or D (right atrium), as described previously [10,12]. Incorrect position was defined as too deep insertion (in the right atrium) or displacement of the catheter either cranially (from subclavian veins) or into subclavian veins (from the internal jugular vein) and necessity to correct CVC tip position by the interventionist.

### Study procedure

**Fig 2:**
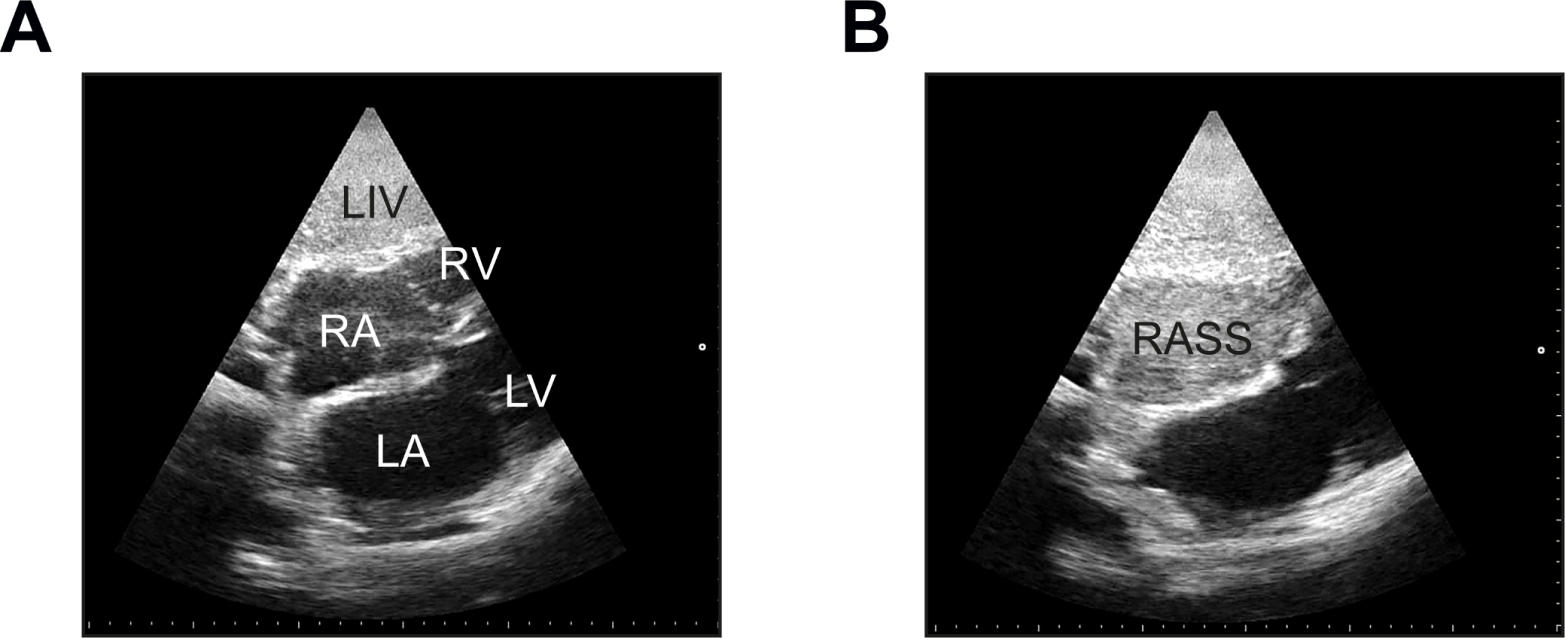
Demonstration of the “rapid atrial swirl sign” (RASS). **(A)** Anatomic structures from a subcostal view. LA, left atrium; LIV, liver; LV, left ventricle; RA, right atrium; RASS, rapid atrial swirl sign; RV, right ventricle. **(B)** Complete opacification of the right atrium after rapid injection of 10 mL of normal saline through the distal hub of the catheter.

Focused echocardiography using subcostal (SC) or apical four-chamber views (4CV) (the index test) was taught individually to all residents participating in the study in a brief 30 to 60 minutes’ session. Echocardiography was performed using a sector probe of either the Esaote MyLab5 or Esaote MyLabGold (Esaote S.p.A., Genova, Italy) US machines. Immediately after CVC placement, a saline flush consisting of 10 mL of normal saline was injected into the distal hub of the CVC by the interventionist while focused echocardiography was conducted by a second resident (**Fig 2**). The exam was recorded in a short video sequence on the hard disk of the US machine and the time required for the procedure was recorded. Appearance of an opacification of the right atrium (RASS) was judged as “immediate” (less than two seconds after injection), “delayed” (appearing more than two seconds after injection) or “absent” as proposed by others [8,10]. Echocardiography was immediately evaluated by the resident placing the catheter and the result (“delayed”, “immediate” or “absent” flush) recorded by one investigator (EM). The examination could be repeated up to three times and catheter position corrected during placement when the saline flush test was indicative of a problem. Overall, a positive RASS (negative screening test for misplacement) translates into a correctly positioned catheter, whereas a delayed or absent flush (negative RASS or positive screening test) implies a potentially misplaced catheter.

### Ethics approval and trial registration

The study was approved by the Medical Ethics Committee at the University Medical Center Goettingen (registry number 11/3/14) on October 8th, 2014 and all patients or their authorized representatives provided written informed consent. The full study protocol was retrospectively registered with ClinicalTrials.gov (number NCT02661607) in June 2015. The reason for retrospectively registering the study was that the study authors were not aware of the recommendation to register diagnostic accuracy studies before this date. The authors confirm that all ongoing and related trials for this intervention are registered. The first patient was included on November 10th, 2014, the last patient was included on April 5th, 2016.

### Statistical methods

Descriptive statistics and statistical testing were performed using GraphPad Prism version 5.0 (GraphPad Software, San Diego California USA, www.graphpad.com), MedCalc Statistical Software version 16.4.3 (MedCalc Software bvbam Ostend, Belgium; www.medcalc.org; 2016), or R version 3.3.1 (R Foundation, Vienna Austria; www.r-project.org). Normal distribution was assessed by quantile-quantile plots and Shapiro-Wilk test. Student’s t-test or Mann-Whitney U test (for non-gaussian data) were used for comparisons between two groups, while measurements in the same patient were analyzed with the corresponding test for paired data. To compare echocardiography with CXR, sensitivity, specificity, positive and negative predictive values and likelihood ratios were calculated with CXR as a reference standard. Sample size estimation is given in detail in Appendix 2. Measurement of the catheter tip in relation to the carina between two independent observers (EM and SW) was analyzed using Bland-Altman plots since continuous variables were assessed. Based on the recommendations for retrospective chart reviews for the assessment of interrater agreement by Kaji et al. [13], a random sample of 30% of echocardiography video recordings was independently evaluated by two investigators and the interrater agreement was calculated using Cohen’s kappa statistic as these were categorical variables. For the duration time of echocardiography, mixed-effect models were used to account for the repeated measurements per sonographer. P-values <0.05 were considered statistically significant.

## Results

### Study cohorts

One patient from the test cohort with a body mass index more than 40 kg/m2 was excluded from the study because echocardiography was not possible to perform despite multiple attempts. The reference cohort included 10 patients, the test cohort included 90 patients. In the test cohort, median age was 68 years, 47 patients were female, 43 were male. The majority (89%) of patients had no respiratory support, 96% of catheters were 3-lumen central venous catheters and the right internal jugular vein was the most frequently assessed insertion site (53.3%), followed by the left internal jugular vein (41.1%). CVC insertion was performed using a dynamic (ultrasound visualization of the vessel throughout the procedure) approach in over 80% of procedures. The subclavian vein was cannulated in only a minority of patients using a landmark approach or sonographic guidance. Characteristics of the reference cohort were similar to the test cohort (**Table 1**), there were no statistically significant differences. No patient had a heart rate below 50 beats per minute (data not shown).

**Table 1:** Description of the study cohorts.

	Reference cohort (n = 10)	Test cohort (n = 90)	
**Median age (min-max)**	70.0 (36–91)	68.0 (19–90)	p = 0.3099
**Female sex**	5 (50%)	47 (52.2%)	p = 0.8414
**ICU**	5 (50%)	38 (42.2%)	p = 0.8713
**Respiratory support**			
none	8 (80%)	80 (89%)	p = 0.7141
NIV	1 (10%)	5 (5.5%)	
IV	1 (10%)	5 (5.5%)	
**CVC type**			
3-lumen	9 (90%)	86 (96%)	p = 0.5654
4-lumen	1 (10%)	3 (3%)	
5-lumen	0 (0%)	1 (1%)	
**CVC insertion site**			
R internal jugular vein	5 (50%)	48 (53.3%)	p = 0.8312
L internal jugular vein	4 (40%)	37 (41.1%)	
R subclavian vein	0 (0%)	2 (2.2%)	
L subclavian vein	1 (10%)	3 (3.3%)	
**CVC insertion US guided**	7 (70%)	74 (82.2%)	p = 0.6102

**Abbreviations used:** CVC: central venous catheter, ICU: intensive care unit, IMC: intermediate care unit, IV: invasive ventilation, L: left, NIV: non-invasive ventilation, R: right, US: ultrasound.

### Central venous catheter assessment using chest radiography and the echocardiographic “rapid atrial swirl sign”

**Fig 3:**
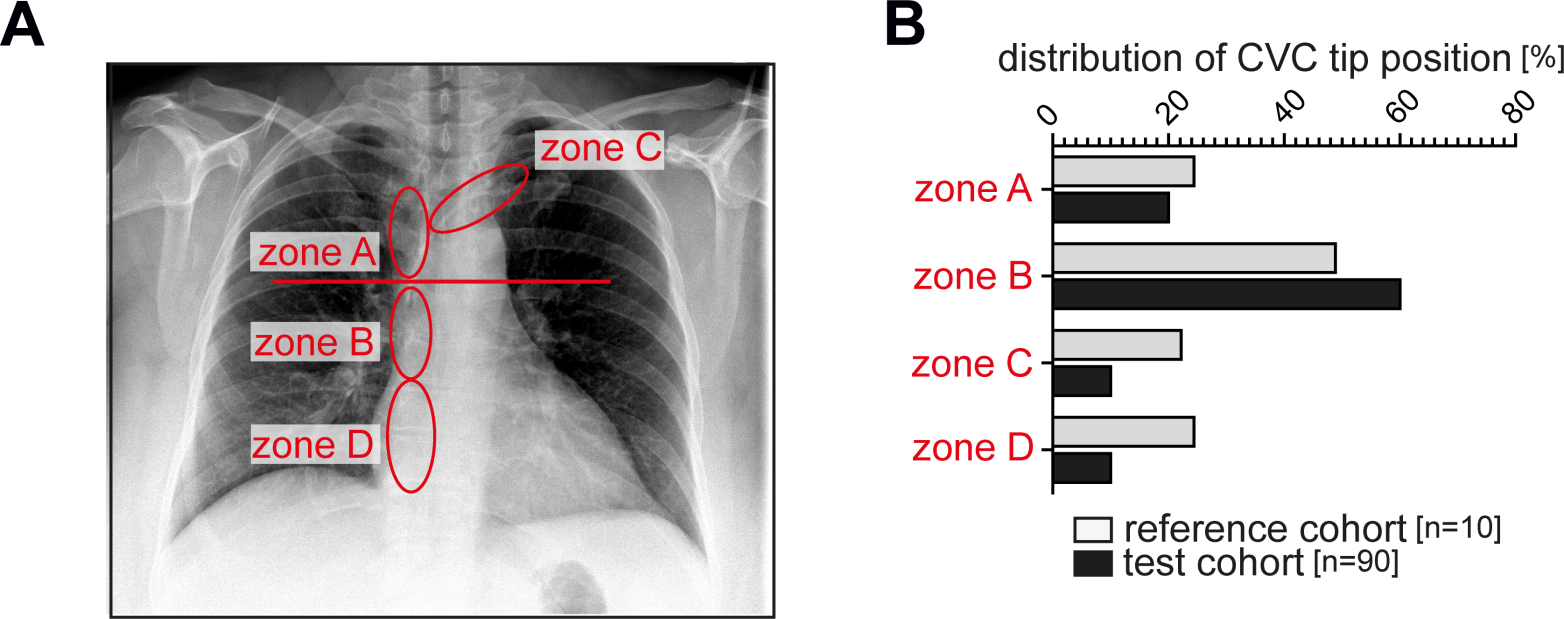
(A) Definition of zones of catheter tip placement on conventional chest radiographs. (B) Relative distribution of the number of placed catheters according to zones A, B, C or D in the reference (white) and test cohort (black).

**Fig 4:**
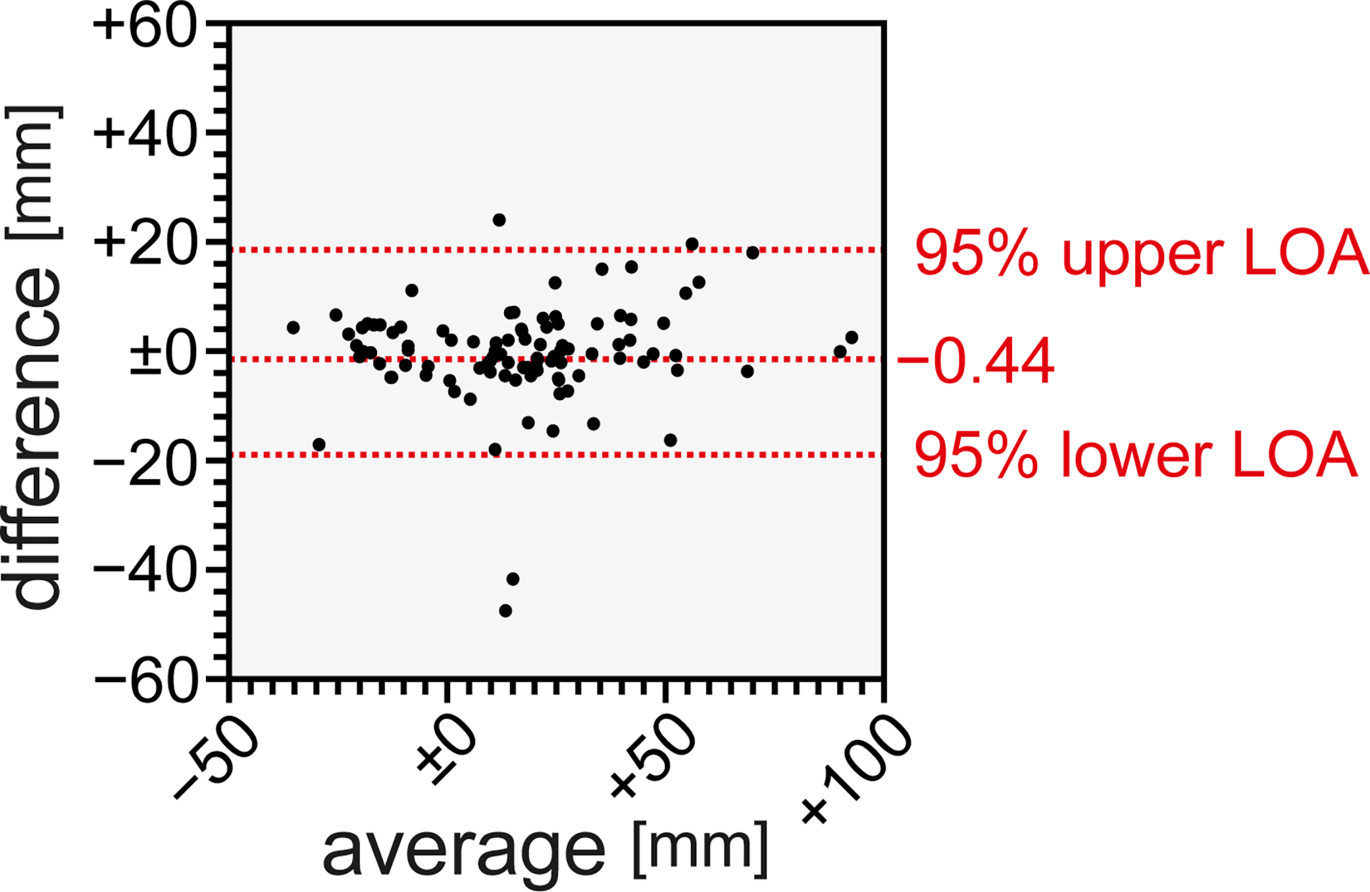
Bland-Altman plot of agreement for catheter tip position assessment between two raters in chest radiographs. **Abbreviations used:** LOA: limit of agreement.

Results of the distribution according to zones A-D are shown in (**Fig 3A and 3B)**. Most catheters were placed in the traditionally recommended zones A and B. Overall, 23 catheters were found to be placed within the area of the right atrium (zone D). 11 of these were retracted at the discretion of the interventionist. Mean difference of average between the measurements of two investigators (EM and SW) are shown in **Fig 3**. Mean difference of average was determined at -0.44 mm (95% limit of agreement from -19.09 to 18.21) for CVC tip height assessment in relation to the carina by two different raters (**Fig 4**). Thus, the measurements did not show any clinically relevant differences.

Focused echocardiography was performed using either a subcostal (SC) or apical four-chamber view (4CV). In the majority of patients (86%), a SC view was accessible, whereas a 4CV was necessary in 15 patients due to anatomical limitations. Overall, CXR showed a misplaced catheter in twelve patients. A positive flush test (flush appearing within two seconds in the right atrium) was present in 83 of 100 patients. A delayed flush was observed in 9 of 100 patients and an absent flush seen in 8 of 100 patients (**Table 2**). For subsequent analyses, both delayed and absent flush tests were judged as “negative” RASS (positive screening test for misplacement).

**Table 2:** Test results of the study cohorts.

	Reference cohort (n = 10)	Test cohort (n = 90)	All patients (n = 100)	
**CXR position correct**	8 (80%)	80 (88.9%)	88 (88%)	p = 0.7583
**Echocardiography**				
Flush immediate	7 (70%)	76 (84.4%)	83 (83%)	p = 0.3265
Flush delayed	1 (10%)	8 (8.9%)	9 (9%)	
Flush absent	2 (20%)	6 (6.7%)	8 (8%)	
**Echocardiography position**				
4CV	4 (40%)	11* (12.2%)	15* (15%)	p = 0.0591
SV	6 /60%)	80 (88.8%)	86 (86%)	

*in one patient, both views were required to obtain a result

**Abbreviations used:** 4CV: four chamber view; CXR: chest radiography; SV: subcostal view.

### Test characteristics of the “rapid atrial swirl sign”

In the reference cohort, where only one experienced investigator performed all exams, sensitivity was 100%, specificity was 87.5%. The negative predictive value was 100%. The positive predictive value for incorrect CXR position with a negative RASS was 66.7%. The overall test characteristics for the test cohort, where 20 different residents performed the examinations are shown in **Table 3**.

**Table 3:** Test characteristics of the “rapid atrial swirl sign” in the test cohort.

	CXR position incorrect	CXR position correct	Total	Test characteristics (n = 90)
**Screening test positive**				Sensitivity 100% (95% CI 69.15%-100.00%) Specificity 95.0% (95% CI 87.69% to 98.62%) LR+: 20.0 (95% CI 7.69 to 51.98) LR-: 0.00 PPV: 71.43% (95% CI 49.03% to 86.66%) NPV: 100%
*RASS absent*	10	4	14	
**Screening test negative**				
*RASS present*	0	76	76	
**Total**	10	80	90	

**Abbreviations used:** CI: 95% confidence interval, CXR: chest radiography, LR+: positive likelihood ratio, LR-: negative likelihood ratio, NPV: negative predictive value, PPV: positive predictive value, RASS: rapid atrial swirl sign.

### Interrater agreement of the “rapid atrial swirl sign”

When using three variables (appearance of saline flush immediate, delayed or absent) Cohen’s kappa showed good agreement with 0.726 (95% confidence interval (CI) [0.488–0.964]). When ratings were dichotomized, e g. an immediately appearing saline flush was considered a negative screening test for CVC misplacement and delayed or absent saline flush considered a positive screening test for CVC misplacement, the interrater agreement was slightly better with Cohen’s kappa of 0.772 (95% CI [0.533–1.0]).

### Comparison of performance time of echocardiography versus chest radiography

**Fig 5:**
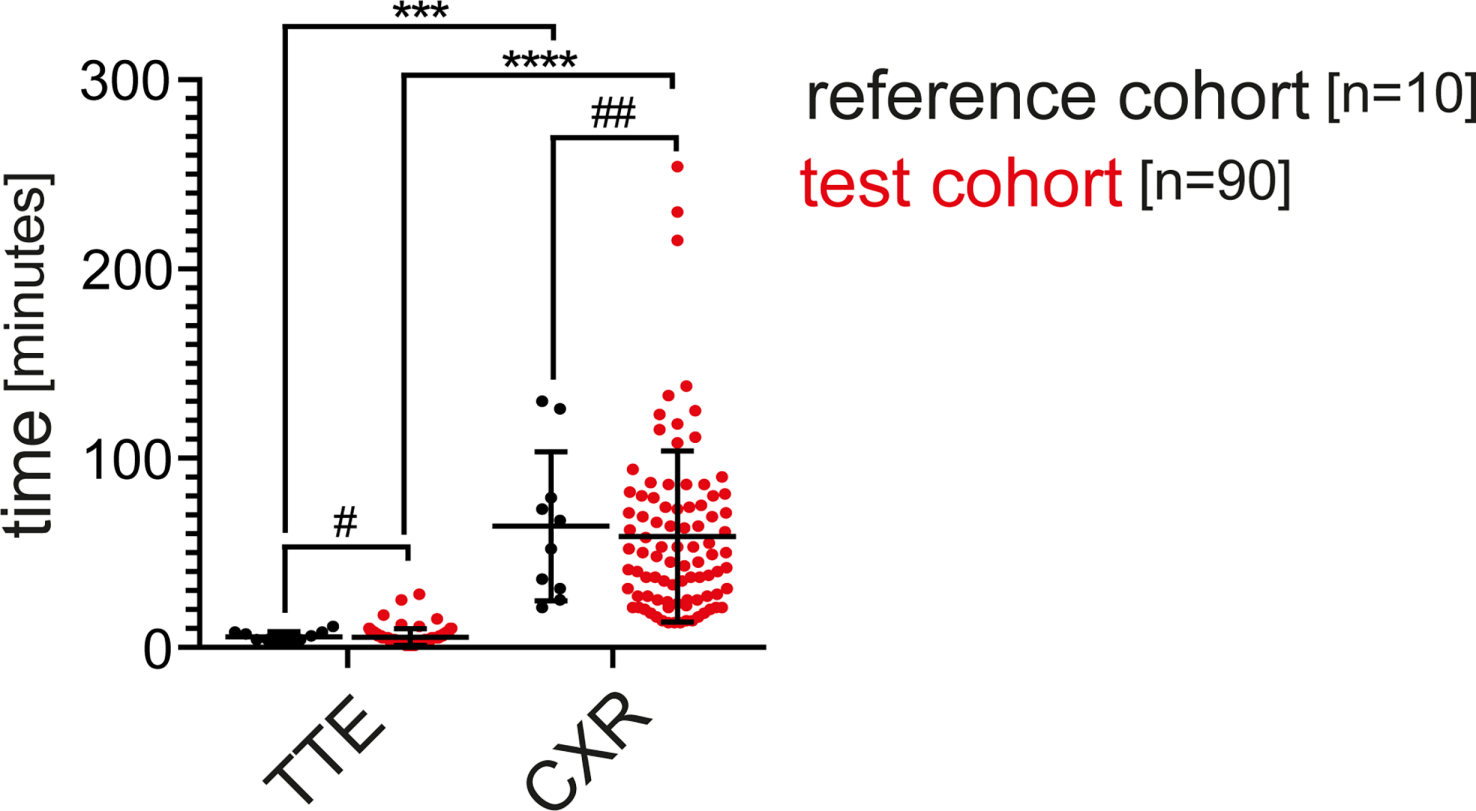
Time needed for transthoracic echocardiography and chest radiography in the reference and test cohorts. #p = 0.4666 ##p = 0.5162 ***p = 0.002 ****p<0.0001 **Abbreviations used:** CXR: chest radiography, TTE: transthoracic echocardiography.

After calling the radiology service, median time for obtaining a CXR was 59.5 and 48.5 minutes in the reference and test cohorts (ranging from 21–130 minutes in the reference cohort and 13–254 minutes in the test cohort). There was no significant difference between these groups (p = 0.5162). The median time needed to obtain a focused echocardiography was 5 minutes in both groups (2–11 minutes in the reference cohort and 1–28 minutes in the test cohort, p = 0,4666), which was significantly shorter than the time needed until a CXR was available (p = 0.002 and p<0.0001) (**Fig 5**).

### Performance and test characteristics of the “rapid atrial swirl sign” in different groups of residents

Throughout the study period, a total of 20 different residents assigned to the ICU and IMC were involved in obtaining data. There was a difference in numbers between the PGY 1 and 2 and the other two (years 3 and 4, years 5 and 6) groups. The first group consisted of 10 different residents with a median number of 2.5 (range 1–6) performed US examinations per resident. The other two groups included 5 different residents, respectively, and the median number of US examinations was slightly higher, but not statistically significant, with a median number of 6 (1–14) and 3 (1–16) tests performed by each resident (**Table 4**).

**Table 4:** Composition of the different groups of residents and overall test characteristics.

	PGY 1/2 (n = 27)	PGY 3/4 (n = 28)	PGY 5/6 (n = 35)
Number of residents	10	5	5
Median number of US examinations per resident (range)	2.5 (1–6)	6 (1–14)	3 (1–16)
Sensitivity	100% (CI 15.81–100%)	100% (CI 47.82–100%)	100% (CI 29.24–100%)
Specificity	96% (CI 79.65–99.9%)	91.43% (CI 76.94–98.2%)	97.06% (CI 84.67–99.93%)
PPV	66.67% (CI 9.43–99.16%)	62.5% (CI 24.49–91.48%)	75% (CI 19.41–99.37%)
NPV	100% (CI 85.75–100%)	100% (CI 89.11–100%)	100% (CI 89.42–100%)
LR+	25 (CI 3.66–170.59)	11.67 (CI 3.95–34.42)	34 (CI 4.93–234.47)
LR-	0	0	0

**Abbreviations used:** CI: 95% confidence interval, LR+: positive likelihood ratio, LR-: negative likelihood ratio, NPV: negative predictive value, PGY: post-graduate year, PPV: positive predictive value.

**Fig 6:**
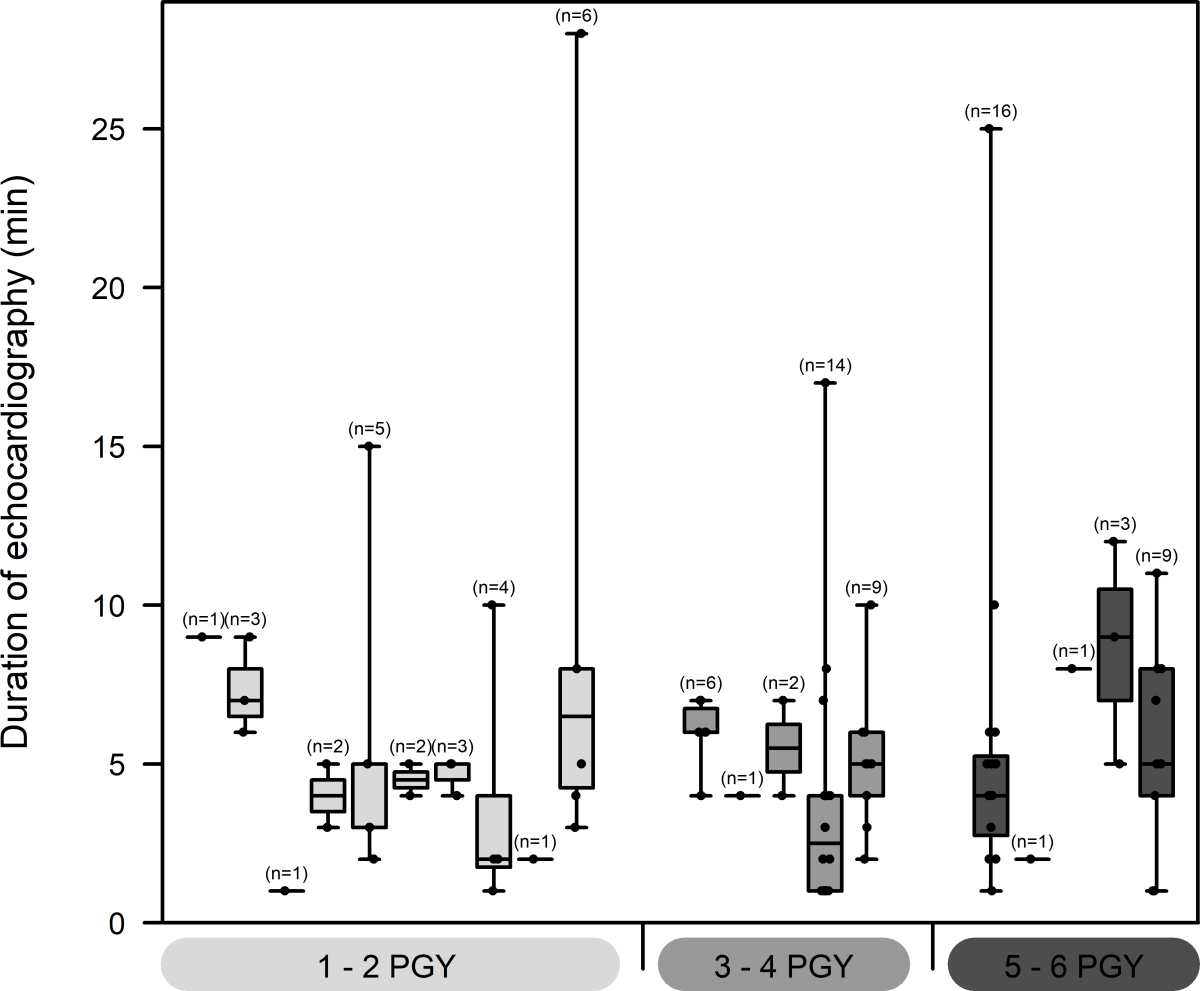
Number and duration of examinations performed at the resident level. The Boxplot shows the distribution and sample sizes of duration of echocardiography measurements in minutes for all 20 sonographers. On the x-axis, sonographers are grouped according to experience (PGY 1/2, 3/4 and 5/6). The boxes show median values and 25% and 75% quantiles with minimum and maximum as whiskers. Additional black dots represent the original data.

Median duration echocardiography of was 5 minutes in the 1–2 PGY group, 4 minutes in the 3–4 PGY group and 5 minutes in the 5–6 PGY group. Mixed effect models did not show any significant effect of PGY. Within subject correlation was found to be close to zero (**Fig 6**), indicating that there are no crucial differences in performance between various sonographers.

All three groups of residents performed a similar number of exams (27, 28 and 35, respectively). Test characteristics were similar among all groups (**Table 4**). The positive predictive value and positive likelihood ratio were highest in the most experienced resident group (75% and 34, respectively).

### Complications of central venous catheterization

In the whole study cohort, there was one pneumothorax (1%) detected by chest radiography, which did not require therapy. No arterial cannulations did occur. Our protocol did not include routine screening for pneumothorax with US. There were twelve misplaced catheters, mostly in zone D, as defined in the methods section. An additional 11 catheters also projected in radiological zone D, but these were left unchanged as per decision of the interventionist placing the catheter and therefore not regarded misplaced as per our definition (see methods section). All catheters were corrected according to the results obtained by echocardiography and chest radiography (either slight retraction or repositioning for aberrant positions). There were no complications associated with the focused echocardiography examination or conventional radiography.

## Discussion

Overall, our study shows that use of the RASS (also named “saline flush test” or “bubble test”) indicates correct CVC tip position with an excellent sensitivity (100%) and specificity (about 94%) independent of investigator experience and after very limited training. We investigated the RASS as diagnostic test in two independent cohorts (reference cohort with ten patients, test cohort with 90 patients) and the performance of 20 differently experienced residents (post-graduate years one to six), which did not differ significantly. Performing the RASS is applicable in most patients, we only excluded one patient with extreme obesity because we were not able to obtain appropriate echocardiographic views. Compared to obtaining chest radiographs, echocardiography was considerably faster (median of 5 minutes vs. 13–254 minutes).

In clinical practice, this would mean that by obtaining a positive echocardiographic RASS, a routine CXR would not be necessary. A negative RASS, to the contrary, suggests a misplaced catheter.

Methodologically, there are situations that might make it a challenge to perform and/or to interpret the examination correctly. Notably, in 17 positive screening tests (negative RASS), five catheters were placed correctly as judged by CXR and therefore left unchanged. In these examinations, difficult anatomical situations might have led to erroneous results.

The optimal position of a CVC tip position has been a matter of debate for many years [12] and traditional teaching has highlighted complications from misplaced catheters, such as sclerosing of central veins, puncture of vessels or rhythm disturbances. Recently, the discussion has extended to dedicated weblog posts [14] concluding that most catheters traditionally regarded as “misplaced” might still be used. In our study, we regarded a catheter as misplaced when it was corrected by the interventionist.

The RASS (or variants thereof) have been studied in different settings and by different specialists. Horowitz *et al*., for example, used the RASS for confirmation of correct venous placement during cardiac catheterization in a pediatric population [11]. Meggiolaro and colleagues have investigated the RASS in a preoperative setting [15]. In contrast to our trial, investigators in the various published studies had undergone a formal US training or had longer expertise in echocardiography [8,10,11,15–19]. Overall, our diagnostic accuracy study confirms results from other, similarly designed studies, which proved the RASS to be a useful test for the confirmation of CVC tip position with enough certainty for clinical practice. Throughout the study, while not formally assessed, we experienced a variety of situations where US was difficult to perform either due to anatomical or logistic circumstances. Image quality is, in fact, the most important limitation in examinations which are difficult to interpret and to perform.

In our ICU and IMC, we treated 954 and 1,222 patients in 2015, respectively. In the ICU, an estimated 90% received a CVC, while in the step-down IMC unit, the frequency was about 50%. Overall, this estimate results in about 1,469 placed CVCs annually in our department. Based on our results, we suggest performing CXR only in those patients with a negative flush test. In our population, these were 17 of 100 patients (**Table 3**), thus potentially reducing routine CXR by more than 80%. Additionally, forgoing routine CXR might be cost-saving. At our institution, performing a chest radiograph in an intensive care setting costs about 18€ (not including costs for personnel), thus reducing the number of radiographs from 1469 to about 294 would lead to a substantial cost reduction (from 26442 € to 5292 € per year).

Our study has several limitations. First, we did not routinely evaluate all patients for the presence of a post-procedural pneumothorax with US as suggested by others [20]. In our view, detecting a pneumothorax by US requires a more formal training to be sufficiently reliable and was not the purpose of this study. Second, routine scanning of all accessible vessels (internal jugular veins, subclavian veins) offers the advantage of possible detection of misplaced catheters immediately after the saline flush test. We decided against this routine scanning, because our primary goal was to compare the test characteristics of echocardiography with CXR and we were primarily interested in the applicability of a simple test by various residents with little training.

Nevertheless, with a negative RASS, we would opt for scanning all veins for misplaced catheters as these could still be corrected during the procedure, thus avoiding additional CXR given the RASS yields a positive result after correction.

Despite the above mentioned limitations, our study has several strengths in addition to other similar studies: Our results show a good interrater agreement, which indicates that a simple decision of saline flush being present or absent can be made with sufficient certainty between different observers even when only video recordings of the echocardiography are available as these might give a slightly different impression of the examination (compared to bedside examinations) and were of varying quality.

Also, we included residents from post-graduate years one to six. Other similarly designed studies often include only a limited number of investigators, which might be very familiar with US procedures. Our study therefore reflects routine clinical situations, where differently experienced physicians are performing ultrasound examinations. The residents included in our study were all familiar with the use of vascular US during placement of the CVCs, however, they had little or no experience at all in transthoracic echocardiography before they were trained.

These results suggest that the test can be equally well performed after a short learning session and all residents were able to identify possible misplaced catheters. There was, however, a varying number of exams performed per resident as a potential source of bias. Some residents performed only one examination while there were others who performed more. In our analysis of the data, however, this varying number did not influence the overall test results significantly. The residents who performed more examinations also required more than the median time of five minutes in some instances.

In addition to be considerably faster (median difference of 45 minutes), echocardiography does not rely on radiation. Medical imaging is the major source of radiation exposure for the general population [21]. A comparison of 14 different countries revealed that Germany was second in annual frequency of x-rays and that diagnostic imaging contributed with 1.5% to the cumulative cancer risk [21]. While the associated risks vary for different modalities, our efforts regarding patient safety should still aim at reducing unnecessary radiation exposure.

## Conclusions

The present study confirms the usefulness of the *“rapid atrial swirl sign”* for the assessment of central venous catheter tip position when compared to routine chest radiography. In our study, radiographs could theoretically be reduced by 80% with substantial reductions in radiation exposure, time and costs. The test can be rapidly and reliably performed by novice sonographers after limited training.

## Supporting information

S1 Table Standard reporting in diagnostic accuracy studies (STARD) checklist. (DOCX)

S1 Text Sample size estimation. (DOCX)

S1 File Study protocol, English. (PDF)

S2 File Study protocol, German. (PDF)
