# Supplementary material for: The "rapid atrial swirl sign" for assessing central venous catheters: Performance by medical residents after limited training
Source: PLoS One. 2018 Jul 16;13(7):e0199345. doi: 10.1371/journal.pone.0199345 (PMC6047781; doi:10.1371/journal.pone.0199345)
Supplement: S1 Table — (DOCX) [file pone.0199345.s001.docx]

**Supporting information file 1. STARD checklist**

| **Section & Topic** | **No** | **Item** | **Manuscript page** |
| --- | --- | --- | --- |
| **TITLE or ABSTRACT** |  |  |  |
|  | 1 | Identification as a study of diagnostic accuracy using at least one measure of accuracy | 1 |
| **ABSTRACT** |  |  |  |
|  | 2 | Structured summary of study design, methods, results, and conclusions | 2 |
| **INTRODUCTION** |  |  |  |
|  | 3 | Scientific and clinical background, including the intended use and clinical role of the index test | 3 |
|  | 4 | Study objectives and hypotheses | 3 |
| **METHODS** |  |  |  |
| *Study design* | 5 | Whether data collection was planned before the index test and reference standard were performed (prospectively) or after (retrospectively) | 4-5 |
| *Participants* | 6 | Eligibility cirteria | 3 |
|  | 7 | On what basis potentially eligible participants were identified | 3 |
|  | 8 | Where and when potentially eligible participants were identified (setting, location and dates) | 3 |
|  | 9 | Whether participants formed a consecutive, random or convenience series | 3 |
| *Test methods* | 10a | Index test, insufficient detail to allow replication | 4 |
|  | 10b | Reference standard, in sufficient detail to allow replication | 4 |
|  | 11 | Rationale for choosing the reference standard (if alternatives exist) | 4 |
|  | 12a | Definition of and rationale for test positivity cut-offs or result categories of the index test, distinguishing between pre-specified from exploratory | 4 |
|  | 12b | Definition of and rationale for test positivity cut-offs or result categories of the reference standard, distinguishing pre-specified from exploratory | 4 |
|  | 13a | Whether clinical information and reference standard results were available to the performers/readers of the index test | 4-5 |
|  | 13b | Whether clinical information and reference standard results were available to the performers/readers of the reference standard | 4-5 |
| *Analysis* | 14 | Methods for estimating or comparing measures of diagnostic accuracy | 5 |
|  | 15 | How indeterminate index test or reference standard results were handled | N/A |
|  | 16 | How missing data on the index test and reference test were handled | N/A |
|  | 17 | Any analyses of variability in diagnostic accuracy, disitnguishing pre-specified from exploratory | 5 |
|  | 18 | Intended sample size and how it was determined | 5, Supplementary file S2 |
| **RESULTS** |  |  |  |
| *Participants* | 19 | Flow of participants, using a diagram | Figure 1 |
|  | 20 | Baseline demographic and clinical charasteristics of participants | 5, Table 1 |
|  | 21a | Distribution of severity of disease in those with the target condition | N/A |
|  | 21b | Distribution of alternative diagnoses in those without the condition | N/A |
|  | 22 | Time interval and any clinical interventions between index test and reference test | 5-6; Figure 4A |
| *Test results* | 23 | Cross tabulation of the index test (or their distribution) by the results of the reference standard | 6; Table 3 |
|  | 24 | Estimates of diagnostic accuracy and their precision | 6; Table 3 |
|  | 25 | Any adverse events from performing the index test or the reference standard | 7 |
| **DISCUSSION** |  |  |  |
|  | 26 | Study limitations, including for potential sources of bias, statistical uncertainty, and generalizability | 7-8 |
|  | 27 | Implications for practice, including the intended use and clinical role of the index test | 7-8 |
| **OTHER INFORMATION** |  |  |  |
|  | 28 | Registration number and name of registry | 4-5 |
|  | 29 | Where the full study protocol can be accessed | 4-5 |
|  | 30 | Sources of funding and other support; role of funders | 1 |
