# Supplementary material for: The "rapid atrial swirl sign" for assessing central venous catheters: Performance by medical residents after limited training
Source: PLoS One. 2018 Jul 16;13(7):e0199345. doi: 10.1371/journal.pone.0199345 (PMC6047781; doi:10.1371/journal.pone.0199345)
Supplement: S1 Text — (DOCX) [file pone.0199345.s002.docx]

**Supporting information file 2. Sample size estimation**

*The estimated sample size needed was calculated as follows*^1^*:*

**Sample size prevalence survey** (Box #1)^2^ **:**

Planning to quantify a specificity of above 80% at a precision of 10% (i.e. 95% confidence interval) we estimated that at least 62 patients with correct CVC placement were required.

**Sample size needed to observe at least n events** (Box #3)^2^ **:**

To obtain 62 patients with correct CVC placement (with a probability of 95%), while assuming that at least 4 out of 5 CVCs can be placed correctly, a total number of 86 patients needed to be included in the study.

Since dropouts may occur, we considered a planned sample size of 90 as appropriate.

**References**

1. Machin D, Campbell MJ, Fayers P, Pinol A. Sampe Size Tables for Clinical Studies. 2nd ed. Blackwell; 1997.
2. Glaziou P. URL: *http://sampsize.sourceforge.net/iface/index.html#prev*
