# Supplementary material for: The "rapid atrial swirl sign" for assessing central venous catheters: Performance by medical residents after limited training
Source: PLoS One. 2018 Jul 16;13(7):e0199345. doi: 10.1371/journal.pone.0199345 (PMC6047781; doi:10.1371/journal.pone.0199345)
Supplement: S1 File — (PDF) [file pone.0199345.s003.pdf]

Prospective study: point-of-care ultrasound versus chest radiography for the assessment of central venous catheter position

Application to the ethics committee of the Faculty of Medicine of the Georg-August-University Göttingen, Germany

Title of the study:
Point of care ultrasound versus chest x-ray for position control of central venous catheter

Responsible project leader:
Prof. Dr. med. Gerhard-Anton Müller

Main investigators:
Eirini Mavropoulou
Dr. med. Peter Korsten

Other physicians:
Prof. Dr. med. M. Koziolek (Attending physician of the Intensive Care Unit 1022), Department of Nephrology and Rheumatology
Prof. Dr. med. O. Gross (Attending physician of the Intermediate Care Unit 1021), Department of Nephrology and Rheumatology
Resident physicians of the Intensive care unit 1022 and Intermediate care unit 1021 of various training levels (2nd to 6th year of education)

Address:
University Medical Center Goettingen
Department of Nephrology and Rheumatology
Robert-Koch-Str. 40
D-37075 Goettingen, Germany
Tel. 0551-39-91374
Fax. 0551-39-10285
Email: irinaki87@windowslive.com, peter.korsten@med.uni-goettingen.de

TABLE OF CONTENTS

1. **Synopsis**
2. **Subject of the investigation**
   - 2.1. Scientific background
   - 2.2. Study objective
3. **Study execution**
   - **3.1. Studiendesign**
   - **3.2. Course of studies**
   - **3.3. Study medication**
4. **Patient recruitment**
5. **Biometry**
6. **Undesirable effects**
7. **Legal and ethical provisions**
   - **7.1. Ethical Committee**
   - **7.2. Pharmaceutical legislation**
   - **7.3. Data protection and archiving**
   - **7.4. Patient insurance**
   - **7.5. Funding**

Attachments

Signatures

Literature

Prospective study: point-of-care ultrasound versus chest radiography for the assessment of central venous catheter position

1. Synopsis

**Title of the study:**	Point of care ultrasound versus X-ray thorax for position control of central venous catheter
**Department:**	Nephrology and Rheumatology, University Medical Center Göttingen
**Aim of study:**	Comparison between ultrasound and conventional radiographs for position assessment of central venous catheters
**Study design:**	Prospective, monocentric study
**Estimated study period:**	October 2014 to June 2015 (or until the targeted patient number is reached)
**Patient cohorts:**	Patients of the Intermediate Care Unit (1021) and Intensive Care Unit (1022), who are supplied with a central venous catheter as part of their intensive medical care
**Patient number:**	IMC patients: n = 50<br>ICU patients: n = 50

2. Aim of the investigation

2.1. Scientific background

The ultrasound-guided placement of central venous catheters (CVCs) is now an established procedure. Numerous studies have shown that the use of ultrasound in this setting reduces the rate of typical complications [1]. This applies both to the placement of catheters via the internal jugular veins as well as via the subclavian vein [2].

For this reason, the use of ultrasound is recommended for the placement of CVCs by national and international guidelines [3].

Chest radiographs after CVC placement are at present the standard procedure for the assessment of correct CVC position [4]. The correct position is defined as position of the catheter tip in the superior vena cava, i.e. at the level of the carina (division of the trachea into the main bronchi).

However, there are some limitations and disadvantages of x-ray imaging:

- The time delay from request to execution in critically ill patients
- The sensitivity for the detection of a pneumothorax is only about 40% [5] for supine patients (which is the standard for ICU and IMC patients).
- The use of radiation

The potential advantages of ultrasound examinations which result from these disadvantages for radiographs are:

- It is always available
- No use of radiation
- Better sensitivity for the detection of pneumothorax (about 90%) [6]
-

Currently, there are no established methods for the correct position determination of the catheter tip by means of ultrasound.

There are few studies dealing with the question of whether an ultrasound examination at the bedside can possibly completely replace chest radiographs for catheter tip position assessment.

Two preliminary studies were presented in April 2013 at the *Annual convention of the American Institute of Ultrasound in Medicine* in New York.

The first study, presented by Dr. Kathleen Calabrese (George Washington University Medical Center, Washington, DC) focused on the question of placement of the central venous catheter. Calabreses' team hypothesized that if the catheter is correctly positioned, swirling (flush) will become visible within one second after injection of 10 cc of normal saline solution (NaCl 0.9%) on the ultrasound image. In order to compare the ultrasound method (in this case the representation of the heart cavities of subxiphoid, echocardiographically) with the chest radiography, the researchers examined 28 patients who received a central venous catheter in an intensive medical setting. After application of 10 cc of NaCl 0.9%, the chest radiographs and ultrasound images were consistent in 100% of the cases [7].

The second study, the *Sonographic Evaluation of Central Line Placement* (SECLIP), used the same methodology to investigate a broad set of questions:

- Can the position of the catheter be confirmed?
- Which complications can be detected?
- What is the time difference?

Dr. Eric Mervis reported the results of the first 55 of 144 patients in the SECLIP observational study. The correct position of the catheter was defined as positioning the tip within 2 cm above the cavo-atrial junction and was also shown by application of 10 cc of NaCl 0.9% solution. In this study, the results were less consistent. The chest radiograph revealed five misplacements, the ultrasound examination only two (91% consistency). No pneumothorax was detected by either method. With regards to the required time, there was a difference of 17 minutes in favor of the ultrasound method, which can mean a faster use of centrally administered drugs (especially catecholamines, larger amounts of fluids) in intensive care patients [8].

2.2. Study objective

The aim of the presented study is to investigate whether the position of a central venous catheter with ultrasound is possible and is equivalent to the standard of care (SOC), postinterventional chest radiographs.

3. Study execution

3.1. Study design

Prospective, monocentric clinical study.

3.2. Course of the study

Within this study, 50 patients each of Intermediate Care (IMC 1021) and intensive care units (ICU 1022), who require a central venous catheter as part of their intensive medical care, will be included.

After an approximately two-hour training of all physicians by the main investigators, an ultrasound examination with a sector probe in the subxiphoid, parasternal or apical (depending on the anathetic conditions) position is carried out after CVC placement in standard technique (internal jugular vein or subclavian vein).

The resident physicians participating in the study are residents of various departments of the Department of Internal Medicine who are rotating in the IMC unit and ICU as part of their residency training. In particular, resident physicians from the 2nd to 6th year (of a 6 year residency) of training are rotating to the respective units .

It is essential that the right atrium is visualized during the examination. Then 10 cc of NaCl 0.9% is applied as a bolus via the CVC. This is not different from the otherwise usual administration of saline solution for checking the intravascular position and flushing the catheter. If the fluid bolus in the right atrium becomes visible within one second of application, a correct position in the superior vena cava is assumed. If the catheter tip is visible in the right atrium, the catheter is primarily incorrectly positioned and is immediately corrected (withdrawn). All patients receive a chest radiograph as is usual practice at our institution. The results of the ultrasound are compared with the results of the chest X-ay.

The examination is carried out immediately after the catheter has been placed. At the same time, the staff of the radiology service is informed by telephone to carry out a portable chest radiography.

The time required for the respective examination is determined by stopping the time from the start of the examination to completion of the echocardiography and until the chest radiography is available for evaluation (see **Fig.1**). The echocardiography will not impede performing the chest radiography, thus not endangering the patients.

The results of the ultrasound examinations are stored as a short video clip (so-called "loop") on the device, so that they are reproducible at all times and are accessible to an examination by a physician who is not himself involved in the ultrasound examination.

The examination itself is recorded in a standard protocol by one of the main investigators (E.M. or P.K.), which also documents, which resident physician carrried out the examination.

We would point out that the conduct of the ultrasound examination will in no way influence the patients' safety or treatment as an echocardiographic examination should be carried out anyways in all intensive care patients for hemodynamic evaluation. Furthermore, standard saline solution is applied for verification of the intravascular position of the CVC and a chest radiograph is also performed as standard of care.

It is not expected to endanger the patients, since the ultrasound examination, after appropriate instruction, even in unexperienced investigators, takes only about 5 minutes: The arrival of the X-ray apparatus to the unit takes approximately 15 minutes. The establishment of the procedure could therefore even reduce the risk to the patients due to the lack of time delay.

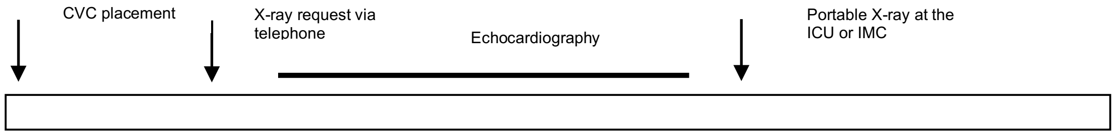

Fig. 1 Timing of events within the study.

3.3. Study medication

NaCl 0.9% 10 cc intravenously.

4. Patient recruitment

A convenience sample of patients in need of a central venous catheter will be recruited for the study. The study will include about 50 patients from the Intermediate Care Unit and 50 patients from the Intensive Care Unit (n=100 in total). Only epidemiological data of all patients are required from the medical records. Further data (e.g. the cause of the inpatient stay or pre-existing conditions) are not collected as these do not play a role for the study.

Patients who are not able to provide informed consent will possibly also be included in the study after determination of their presumed will, since in intensive care, a medically necessary placement a central venous catheter without the possibility of written informed consent from the patient or his legal representative is commonly encountered. The presumed will of the patient who is not able to provide written informed consent is assessed by the treating physician, for example, from the patient's will or by family members and documented in a special form. A subsequent explanation of the participation in a scientific study will take place at the earliest possible time point by the patient or an appointed legal guardian.

Often it is the patients who are not able to provide written informed consent who need to be provided by intensive care procedures, such as CVC placement, immediately. The aim of the study is, among other things, to establish a non-invasive, easy and reliable bedside procedure, which can be carried out without delay and by the staff caring for the patient.

Inclusion criteria:
- Age: 18 years or older.

Exclusion Criteria:
- None.

5. Biometry

The results will be evaluated in the Department of Nephrology and Rheumatology of the University Medical Center Göttingen. A statistical evaluation of the collected data for the comparative evaluation of the ultrasound with chest radiographs will be performed.

6. Adverse events

No adverse effects are expected which go beyond the usual possible complications of a CVC placement since only standard procedures are applied.

7. Legal and ethical provisions

7.1 Ethics Committee

An ethics vote is requested for the planned prospective study.

7.2 Federal Drug Law

Not applicable.

7.3 Data protection and archiving

The study complies with all applicable data protection regulations. For this purpose, patient data are correctly pseudonymized in a database (according to BDSG §3 Abs.6a). The imaging procedures are collected for the evaluation of the clinical data. The data is collected and evaluated exclusively in a pseudonymized form. In this regard, the mentioned patient data are documented on a form sheet. The collected data are stored in study folders and on electronic data carriers in the Department of Nephrology and Rheumatology. A re-identification of patients is only possible for the responsible project investigator and the investigators who are subjected to confidentiality.
The study patient is informed that he / she can withdraw from the clinical study at any time.

All personal data shall be kept for at least 10 years after completion or termination of the study. This data will be deleted if there are no statutory or retention periods.

7.4 Patient insurance

Not applicable

7.5 Funding

The prospective data analysis is neither financed by the industry nor by another sponsor.

7.6 Revocation of consent

Participation in the study is voluntary and can be revoked at any time without giving reasons. The subsequent cancellation of the participation includes the destruction of all collected data and the deletion of all data.

Signatures

______________________	______________________
Place, date	Prof. Dr. med. Gerhard-Anton Müller<br>Head of the Department of<br>Nephrology and Rheumatology
______________________	______________________
Place, date	Eirini Mavropoulou<br>Department of Gastroenterology and<br>Gastrointestinal Oncology
______________________	______________________
Place, date	Dr. med. Peter Korsten<br>Department of Nephrology and<br>Rheumatology

Literature:

[1] Mehta N, Valesky WW, Guy A, Sinert R. Systematic review: is real-time ultrasound-guided central line placement by ED physicians more successful than the traditional landmark approach? Emerg Med J. 2013 May, 30 (5): 355-9. Doi: 10.1136 / emermed-2012-201230. Epub 2012 Jun 26. Review.

[2] Milone M, Di Minno G, Di Minno MN, Salvatore G, Iacovazzo C, Policastro C, Milone F. The real effectiveness of ultrasound guidance in subclavian venous access. Ann Ital Chir., 2010 Sep-Oct; 81 (5): 331-4.

[3] AIUM PRACTICE GUIDELINES-Use of the Ultrasound to Guide Vascular Access Procedures, Effective April 1, 2012.
http://www.aium.org/resources/guidelines/usgva.pdf

[4] American Society of Anesthesiologists Task Force on Central Venous Access, Rupp SM, Apple Tree JL, Blitt C, Caplan RA, Connis RT, Domino KB, Fleisher LA, Grant S, Mark JB, Morray JP, Nickinovich DG, Tung A. For more information, please visit http://www.socialis.org/. Anesthesiology. 2012 Mar; 116 (3): 539-73. Doi: 10.1097 / ALN.0b013e31823c9569.

[5] Alrajab S, Youssef AM, Batteries NI, Caldito G. Pleural ultrasonography versus chest radiography for the diagnosis of pneumothorax: review of the literature and meta-analysis. Crit Care. 2013 Sep 23, 17 (5): R208.

[6] Alrajhi K, Woo MY, Vaillancourt C. Test characteristics of ultrasonography for the detection of pneumothorax: a systematic review and meta-analysis. Chest. 2012 Mar; 141 (3): 703-8. Doi: 10.1378 / chest.11-0131. Epub 2011 Aug 25.

[7] Liu Y, Calabrese K., Ajmera K. et al. Bedside cardiac ultrasound examination to confirm central venous catheter placement. American Institute of Ultrasound in Medicine (AIUM) 2013 Annual Convention; April 9, 2013; New York, NY. Abstract 1540969.

[8] Mervis E., Turner E., Chiem A., et al. Point-of-care ultrasound: Sonographic evaluation of central line placement. American Institute of Ultrasound in Medicine (AIUM) 2013 Annual Convention; April 9, 2013; New York, NY. Abstract 1540557.
