# Supplementary material for: The "rapid atrial swirl sign" for assessing central venous catheters: Performance by medical residents after limited training
Source: PLoS One. 2018 Jul 16;13(7):e0199345. doi: 10.1371/journal.pone.0199345 (PMC6047781; doi:10.1371/journal.pone.0199345)
Supplement: S2 File — (PDF) [file pone.0199345.s004.pdf]

Antrag an die Ethikkommission der Medizinischen Fakultät der Georg-August-Universität Göttingen

Studientitel:

Point of care Ultraschall versus Röntgen-Thorax zur Lagekontrolle zentralvenöser Katheter

Verantwortlicher Projektleiter:

Prof. Dr. med. Gerhard-Anton Müller

Hauptuntersucher:

Eirini Mavropoulou

Dr. med. Peter Korsten

Weitere durchführende Ärzte:

Oberarzt Prof. Dr. med. M. Koziolek (Oberarzt der Intensivstation 1022), Klinik für Nephrologie und Rheumatologie

Oberarzt Prof. Dr. med. O. Gross (Oberarzt der Intermediate Care Station 1021), Klinik für Nephrologie und Rheumatologie

Ärzte der Intensivstation 1022 und IMC-Station 1021 verschiedener Ausbildungsstufen (2. Bis 6. Weiterbildungsjahr)

Adresse:

Universitätsmedizin Göttingen
Klinik für Nephrologie und Rheumatologie
Robert-Koch Straße 40
37075 Göttingen
Tel. 0551-39-91374
Fax. 0551-39-10285
Email: irinaki87@windowslive.com, peter.korsten@med.uni-goettingen.de

INHALTSVERZEICHNIS

1. **Synopsis**
2. **Gegenstand der Untersuchung**
   - 2.1 Wissenschaftlicher Hintergrund
   - 2.2 Studienziel
3. **Studiendurchführung**
   - 3.1 Studiendesign
   - 3.2 Studienablauf
   - 3.3 Studienmedikation
4. **Patientenrekrutierung**
5. **Biometrie**
6. **Unerwünschte Wirkungen**
7. **Rechtliche und ethische Bestimmungen**
   - 7.1 Ethisches Komitee
   - 7.2 Arzneimittelgesetz
   - 7.3 Datenschutz- und –archivierung
   - 7.4 Patientenversicherung
   - 7.5 Förderung

Anhang

Unterschriften

Literatur

1. Synopsis

**Titel der Studie:**	*Point of care* Ultraschall versus Röntgen-Thorax zur Lagekontrolle zentralvenöser Katheter
**Klinik:**	Nephrologie und Rheumatologie, Universitätsmedizin Göttingen
**Studienziel:**	Vergleich zwischen Ultraschall und konventionellem Röntgen zur Lagekontrolle zentralvenöser Katheter
**Studiendesign:**	Prospektive, monozentrische Studie
**Studienzeitraum:**	Oktober 2014 bis Juni 2015 (oder bis die Patientenzahl erreicht ist)
**Patientenkollektiv:**	Patienten von Intermediate Care Unit (1021) und Intensive Care Unit (1022), die im Rahmen der intensivmedizinischen Versorgung mit einem zentralvenösen Katheter versorgt werden
**Patientenzahl:**	IMC Patienten: n=50<br>ICU Patienten: n=50

2. Gegenstand der Untersuchung

2.1 Wissenschaftlicher Hintergrund

Die ultraschallgestützte Anlage von zentralvenösen Kathetern (ZVK) ist mittlerweile ein etabliertes Verfahren. In zahlreichen Studien ist nachgewiesen worden, dass die Verwendung von Ultraschall bei der Anlage die Rate typischer Komplikationen senkt [1]. Dies gilt sowohl für die Anlage von Kathetern über die Vv. jugulares internae als auch für die Vv. subclaviae [2].

Aus diesem Grund ist die Verwendung von Ultraschall bei der Anlage von ZVK in nationalen und internationalen Leitlinien empfohlen [3].

Zur Lagekontrolle von ZVK sind Röntgenaufnahmen des Thorax aktuell der Standard [4]. Die korrekte Lage wird hier definiert als Position der Katheterspitze in der Vena cava superior, d. h. auf Höhe der Carina (Aufteilung der Trachea in die Hauptbronchien).

Es gibt jedoch einige Einschränkungen und Nachteile von Röntgenaufnahmen:

- Die Zeitverzögerung von Anforderung bis zur Durchführung bei kritisch kranken Patienten
- Die Sensitivität zur Detektion eines Pneumothorax liegt bei Liegendaufnahmen (was der Standard ist bei Intensiv- und IMC-Patienten) nur bei etwa 40% [5].
- Die (wenn auch geringe) Strahlenbelastung

Die potentiellen Vorteile von Ultraschalluntersuchungen ergeben sich aus diesen Nachteilen für Röntgenaufnahmen:

- Immer verfügbar
- Keine Strahlenbelastung
- Bessere Sensitivität zur Detektion eines Pneumothorax (etwa 90%) [6].

Aktuell ist kein Verfahren zur korrekten Lagebestimmung der Katheterspitze mittels Ultraschall etabliert.

Es gibt wenige Studien, die sich mit der Frage beschäftigen, ob die Ultraschalluntersuchung am Patientenbett möglicherweise die Röntgenkontrolle gänzlich ersetzen kann.

Zwei vorläufige Studien im April 2013 bei der *Annual convention* des *American Institute of Ultrasound in Medicine* in New York vorgestellt.

Die erste Studie, präsentiert von Dr. Kathleen Calabrese (George Washington University Medical Center, Washington, DC) konzentrierte sich auf die Frage

der Platzierung des zentralvenösen Katheters. Calabreses Team stellte die Hypothese auf, dass bei korrekter Katheterlage eine Verwirbelung (sog. "*flush*") innerhalb einer Sekunde nach Injektion von 10 mL Kochsalzlösung (NaCl 0,9%) auf dem Ultraschallbild sichtbar wird. Um die Ultraschallmethode (in diesem Fall Darstellung der Herzhöhlen von subxiphoidal, d. h. echokardiographisch) mit dem Röntgen-Thorax zu vergleichen, haben die Forscher 28 Patienten untersucht, die einen zentralvenösen Katheter in einem intensivmedizinischen Setting bekommen haben. Nach Applikation von 10 mL NaCl 0.9% zeigte sich eine Übereinstimmung mit dem Röntgenbild in 100% der Fälle [7].

Die zweite Studie, *Sonographic Evaluation of Central Line Placement (SECLIP)*, benutzte die gleiche Methodologie um eine ausgedehnte Reihe von Fragen zu untersuchen:

- Kann die Position des Katheters bestätigt werden?
- Welche Komplikationen können detektiert werden?
- Wie ist der zeitliche Unterschied?

Dr. Eric Mervis berichtete die Ergebnisse der ersten 55 von 144 Patienten in der SECLIP Beobachtungsstudie. Die korrekte Lage des Katheters wurde als Positionierung der Spitze innerhalb 2 cm oberhalb des cavo-atrialen Überganges definiert und wurde ebenfalls durch Applikation von 10 mL NaCl 0.9%-Lösung dargestellt. Hier waren die Ergebnisse weniger gut übereinstimmend. Die Röntgenaufnahme deckte fünf Fehlpositionen auf, die Ultraschalluntersuchung lediglich zwei (91% Übereinstimmung). Es wurde mit beiden Methoden kein Pneumothorax festgestellt. Bezüglich des Zeitfaktors gab es eine Differenz von 17 Minuten zu Gunsten der Ultraschallmethode, was bei Intensivpatienten eine schnellere Nutzung zentralvenös zu applizierender Pharmaka (vor allem Katecholamine, größere Mengen Flüssigkeit) bedeuten kann [8].

2.2 Studienziel

Ziel der vorgelegten Studie ist es, zu untersuchen, ob die Lagebestimmung eines zentralvenösen Katheters mit Ultraschall möglich ist und gleichwertig zum Standard of care (SOC), der postinterventionellen Röntgenaufnahme.

3. Studiendurchführung

3.1 Studiendesign

Es handelt sich um eine prospektive, monozentrische klinische Studie.

3.2 Studienablauf

Im Rahmen der Studie werden je 50 Patienten der Intermediate Care (IMC 1021) und Intensivstationen (ICU 1022) untersucht, die im Rahmen der intensivmedizinischen Behandlung einen zentralvenösen Katheter benötigen.

Nach einem etwa zweistündigen Training aller diensthabenden Ärzte durch die Studienärzte wird nach Anlage des ZVK in Standardtechnik (V. jugularis interna oder V. subclavia) eine Ultraschalluntersuchung mit einem Sektorschallkopf von subxiphoidal, parasternal oder apikal durchgeführt (je nach anatonischen Gegebenheiten).

Bei den diensthabenden Ärzten handelt es sich um im Rahmen der Rotation eingesetzte Assistenzärzte verschiedener Kliniken des Zentrums Innere Medizin. Hier sind Assistenzärzte vom 2. bis 6. Weiterbildungsjahr eingesetzt.

Bei der Untersuchung ist es essentiell, dass der rechte Vorhof dargestellt wird. Nun werden 10 mL NaCl 0.9% als Bolus über den ZVK appliziert. Dies unterscheidet sich nicht von der sonst auch üblichen Verabreichung von Kochsalzlösung zur Überprüfung der intravasalen Lage. Kommt der Flüssigkeitsbolus im rechten Vorhof innerhalb eine Sekunde nach Applikation zur Darstellung, geht man von der korrekten Position in der V. cava superior aus. Ist die Katheterspitze im rechten Vorhof sichtbar, ist der Katheter primär falsch positioniert und wird sofort korrigiert. Alle Patienten erhalten einen Röntgen-Thorax, wie sonst auch üblich. Die Ergebnisse des Ultraschalls werden mit den Ergebnissen des Röntgens verglichen.

Die Untersuchung wird unmittelbar nach Anlage des Katheters durchgeführt, standardmäßig wird zu diesem Zeitpunkt auch das Personal der Röntgenabteilung zur Durchführung einer mobilen Thoraxaufnahme telefonisch benachrichtigt.

Der Zeitbedarf der jeweiligen Untersuchung wird durch Stoppen der Zeit ermittelt von Beginn der Untersuchung bis entweder Abschluss der Echokardiographie oder längstens bis Eintreffen des Röntgenapparates auf der Intensiv- oder IMC-Station (s. Abb.1). Eine Gefährdung der Patienten durch verzögerte Durchführung der Standardröntgenaufnahme findet nicht statt.

Die Ergebnisse der Ultraschall-Untersuchungen werden in Video-Form (sog. „loop“) am Gerät gespeichert, so dass sie jederzeit reproduzierbar sind und einer Auswertung durch einen nicht selbst an der Ultraschalluntersuchung beteiligen Arzt zugänglich sind.

Die Untersuchung selbst wird durch einen der Hauptuntersucher (E.M. oder P.K.) in einem Standardprotokoll festgehalten, hierbei wird auch dokumentiert, welcher Arzt die Untersuchung durchgeführt hat.

Wir weisen darauf hin, dass die Durchführung der Ultraschalluntersuchung in keiner Weise die Patientensicherheit oder Behandlung beeinflussen wird, da eine echokardiographische Untersuchung ohnehin bei allen Intensivpatienten zur hämodynamischen Evaluation erfolgen sollte. Des Weiteren wird standardmäßig Kochsalz zur allgemeinen Prüfung der intravasalen Lage appliziert und ebenfalls ein Röntgen-Thorax standardmäßig durchgeführt.

Eine Gefährdung der Patienten ist u. E. nicht zu erwarten, da die Ultraschalluntersuchung, nach entsprechender Unterweisung, auch bei unerfahrenen Untersuchern nur ca. 5 Minuten dauert, bis zum Eintreffen des Röntgenapparates auf Station vergehen erfahrungsgemäß etwa 15 Minuten. Durch die Etablierung des Verfahrens könnte daher die Gefährdung der Patienten durch den *fehlenden Zeitverzug* sogar reduziert werden.

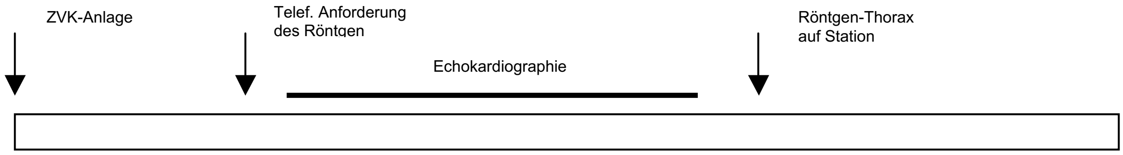

Abb.1 Zeitlicher Ablauf der Studie

3.3 Studienmedikation

NaCl 0,9% 10 ml intravenös.

4. Patientenrekrutierung

Es werden prospektiv alle Patienten, die einen zentralvenösen Katheter benötigen berücksichtigt. In die Studie werden 50 Patienten von der Intermediate Care Unit und 50 Patienten von der Intensive Care Unit eingeschlossen. Aus den Patientenakten wird lediglich das Geburtsjahr der Patienten für die Auswertung benötigt. Weitere Daten (z. B. die Ursache für den stationären Aufenthalt oder Vorerkrankungen) werden nicht erhoben, da diese für die Fragestellung keine Rolle spielen.

Nicht-einwilligungsfähige Patienten sollten nach Feststellung des mutmaßlichen Willens ebenfalls in die Studie eingeschlossen werden, da sich im intensivmedizinischen Bereich regelhaft die medizinisch gebotene Notwendigkeit der Anlage eines zentralvenösen Katheters ohne die Möglichkeit der schriftlichen Einwilligung durch den Patienten oder dessen gesetzlichen Vertreter ergibt. Der mutmaßliche Willen des nicht-einwilligungsfähigen Patienten wird vom Arzt zum Beispiel aus der Patientenverfügung oder durch die Angehörigen überprüft und in einem speziellen Formular dokumentiert. Eine nachträgliche Aufklärung über die Teilnahme an einer wissenschaftlichen Studie findet zum nächstmöglichen

Zeitpunkt durch den Patienten bzw. inzwischen bestellten gesetzlichen Betreuer statt.

Oftmals sind es gerade die nicht-einwilligungsfähigen Patienten, die schneller versorgt werden müssen. Die Studie verfolgt unter Anderem das Ziel, ein nicht-invasives und bettseitiges Verfahren zu etablieren, das *ohne Zeitverzug* und durch das behandelnde Personal selbst durchgeführt werden kann.

Einschlusskriterien:

- Alter: ab 18 Jahre.

Ausschlusskriterien:

- Keine.

5. Biometrie

Die Ergebnisse werden in der Klinik für Nephrologie und Rheumatologie der Universitätsmedizin Göttingen ausgewertet. Es erfolgt eine statistische Auswertung der erhobenen Daten zur vergleichenden Beurteilung des Ultraschalls mit dem Röntgen.

6. Unerwünschte Wirkungen

Es sind keine unerwünschten Wirkungen zu erwarten, die über die üblichen möglichen Komplikationen einer ZVK-Anlage hinausgehen, da lediglich Standardverfahren angewendet werden.

7. Rechtliche und ethische Bestimmungen

7.1 Ethisches Komitee

Ein Ethikvotum ist für die geplante prospektive Studie beantragt.

7.2 Arzneimittelgesetz

Entfällt.

7.3 Datenschutz und Archivierung

Im Rahmen der Studie werden alle geltenden datenschutzrechtlichen Vorschriften eingehalten. Dazu werden die Patientendaten in einer Datenbank ordnungsgemäß pseudonymisiert (nach BDSG §3 Abs.6a). Für die Auswertung der klinischen Daten wird die bildgebende Diagnostik erhoben.

Die Datenerfassung und –auswertung erfolgt ausschließlich in pseudonymisierter Form. Diesbezüglich werden die genannten Patientendaten auf einem Formblatt dokumentiert. Die erhobenen Daten werden in Studienordner und auf elektronischen Datenträgern in der Klinik für Nephrologie und Rheumatologie gespeichert. Eine Re-Identifizierung der Patienten ist nur für den verantwortlichen Projektleiter und den Prüfarzt, die der Schweigepflicht unterliegen, möglich.
Der/die Studienpatient/in wird darüber aufgeklärt, dass er/sie jederzeit die Teilnahme an der klinischen Studie beenden kann.

Die personenbezogenen Daten werden nach Beendigung oder Abbruch der Prüfung mindestens 10 Jahre aufbewahrt. Danach werden diese Daten gelöscht, soweit nicht gesetzliche oder satzungsgemäße Aufbewahrungsfristen entgegenstehen.

7.4 Patientenversicherung

Entfällt.

7.5 Förderung

Die prospektive Datenanalyse wird weder durch die Industrie noch durch einen anderen Sponsor finanziert.

7.6 Widerruf der Einwilligung

Die Teilnahme an der Studie ist freiwillig und kann jederzeit ohne Angabe von Gründen widerrufen werden. Der spätere Widerruf der Teilnahme schließt die Vernichtung aller erhobenen Daten und die Löschung Ihrer Daten ein.

Unterschriften

_________________ _____________________________

Ort, Datum — Prof. Dr. med. Gerhard-Anton Müller

Direktor der Klinik für Nephrologie

und Rheumatologie

_________________ _____________________________

Ort, Datum — Eirini Mavropoulou

Klinik für Gastroenterologie und Endokrinologie

_________________ _____________________________

Ort, Datum — Dr. med. Peter Korsten

Klinik für Nephrologie und Rheumatologie

Literatur:

[1] Mehta N, Valesky WW, Guy A, Sinert R. Systematic review: is real-time ultrasonic-guided central line placement by ED physicians more successful than the traditional landmark approach? *Emerg Med J.* 2013 May;30(5):355-9. doi: 10.1136/emermed-2012-201230. Epub 2012 Jun 26. Review.

[2] Milone M, Di Minno G, Di Minno MN, Salvatore G, Iacovazzo C, Policastro C, Milone F. The real effectiveness of ultrasound guidance in subclavian venous access. Ann Ital Chir. 2010 Sep-Oct;81(5):331-4.

[3] AIUM PRACTICE GUIDELINES-Use of the Ultrasound to Guide Vascular Access Procedures, Effective April 1, 2012. http://www.aium.org/resources/guidelines/usgva.pdf

[4] American Society of Anesthesiologists Task Force on Central Venous Access, Rupp SM, Apfelbaum JL, Blitt C, Caplan RA, Connis RT, Domino KB, Fleisher LA, Grant S, Mark JB, Morray JP, Nickinovich DG, Tung A. Practice guidelines for central venous access: a report by the American Society of Anesthesiologists Task Force on Central Venous Access. Anesthesiology. 2012 Mar;116(3):539-73. doi: 10.1097/ALN.0b013e31823c9569.

[5] Alrajab S, Youssef AM, Akkus NI, Caldito G. Pleural ultrasonography versus chest radiography for the diagnosis of pneumothorax: review of the literature and meta-analysis. Crit Care. 2013 Sep 23;17(5):R208.

[6] Alrajhi K, Woo MY, Vaillancourt C. Test characteristics of ultrasonography for the detection of pneumothorax: a systematic review and meta-analysis. *Chest.* 2012 Mar;141(3):703-8. doi: 10.1378/chest.11-0131. Epub 2011 Aug 25.

[7] Liu Y, Calabrese K., Ajmera K. et al. Bedside cardiac ultrasound examination to confirm central venous catheter placement. *American Institute of Ultrasound in Medicine (AIUM) 2013 Annual Convention*; April 9, 2013; New York, NY. Abstract 1540969.

[8] Mervis E., Turner E., Chiem A., et al. Point-of-care ultrasound: Sonographic evaluation of central line placement. *American Institute of Ultrasound in Medicine (AIUM) 2013 Annual Convention*; April 9, 2013; New York, NY. Abstract 1540557.
